# Personality traits and career choices among physicians in Finland: employment sector, clinical patient contact, specialty and change of specialty

**DOI:** 10.1186/s12909-018-1155-9

**Published:** 2018-03-27

**Authors:** Sari Mullola, Christian Hakulinen, Justin Presseau, David Gimeno Ruiz de Porras, Markus Jokela, Taina Hintsa, Marko Elovainio

**Affiliations:** 1 Faculty of Educational Sciences, University of Helsinki, (Siltavuorenpenger 5 A), P.O. Box 9, 00014 Helsinki, Finland; 2 National Center for Children and Families, Teachers College Columbia University, Thorndike Hall 525 West 120th Street, Box 39, New York, NY 10027 USA; 3 Department of Psychology and Logopedics, Medical Faculty, University of Helsinki, Helsinki, Finland; 4 Institute for Health and Welfare, P.O. Box 30, 00370 Helsinki, Finland; 5 Clinical Epidemiology Program, Ottawa Hospital Research Institute, 501 Smyth Road, Ottawa, K1H 8L6 Canada; 6 School of Epidemiology and Public Health, University of Ottawa, 600 Peter Morand Crescent, Ottawa, K1G 5Z3 Canada; 7 School of Public Health, The University of Texas Health Science Center at Houston, San Antonio, TX 78229 USA

**Keywords:** Medical career, Medical specialty, Personality traits, Person-job fit, Career counseling, Medical education

## Abstract

**Background:**

Personality influences an individual’s adaptation to a specific job or organization. Little is known about personality trait differences between medical career and specialty choices *after* graduating from medical school when actually practicing different medical specialties. Moreover, whether personality traits contribute to important career choices such as choosing to work in the private or public sector or with clinical patient contact, as well as change of specialty, have remained largely unexplored. In a nationally representative sample of Finnish physicians (*N* = 2837) we examined how personality traits are associated with medical career choices *after* graduating from medical school, in terms of employment sector, patient contact, medical specialty and change of specialty.

**Methods:**

Personality was assessed using the shortened version of the Big Five Inventory (S-BFI). An analysis of covariance with posthoc tests for pairwise comparisons was conducted, adjusted for gender and age with confounders (employment sector, clinical patient contact and medical specialty).

**Results:**

Higher *openness* was associated with working in the private sector, specializing in psychiatry, changing specialty and not practicing with patients. Lower openness was associated with a high amount of patient contact and specializing in general practice as well as ophthalmology and otorhinolaryngology. Higher *conscientiousness* was associated with a high amount of patient contact and specializing in surgery and other internal medicine specialties. Lower conscientiousness was associated with specializing in psychiatry and hospital service specialties. Higher *agreeableness* was associated with working in the private sector and specializing in general practice and occupational health. Lower agreeableness and *neuroticism* were associated with specializing in surgery. Higher *extraversion* was associated with specializing in pediatrics and change of specialty. Lower extraversion was associated with not practicing with patients.

**Conclusions:**

The results showed distinctive personality traits to be associated with physicians’ career and specialty choices *after* medical school independent of known confounding factors. Openness was the most consistent personality trait associated with physicians’ career choices in terms of employment sector, amount of clinical patient contact, specialty choice and change of specialty. Personality-conscious medical career counseling and career guidance during and after medical education might enhance the person-job fit among physicians.

## Background

Training a physician at medical school and in a specialization requires a substantial investment of resources, which ultimately benefits patient and population health. However, as shortages in medical staff continue to be reported worldwide [1, 2], as evidenced by absenteeism, stress and turnover among physicians [3, 4], it is imperative to ensure the correct fit between physicians and their chosen careers and specialties.

Although many factors influence a physician’s career and specialty choice [5–7], personality traits have been suggested to be among the most important individual-level determinants [5, 8–15]. Person-job fit theory [16] postulates that personality traits are an important factor determining how an individual will adapt to a specific job or organization. Personality refers to individuals’ affective, experiential and motivational characteristics that reflect their values, attitudes and coping strategies [17]. A large volume of empirical studies provides convincing evidence of the importance of personality in predicting the person-job fit of physicians-in-training [18]. Previous studies examining the association between personality traits and medical careers have focused on medical students [5, 14, 19–26] and a few specialties, mainly surgery [19, 20, 23, 27] and psychiatry [20–23, 27], with partly inconsistent findings [5, 19, 21–23, 27]. Medical students preferring surgery over other specialties have shown higher extraversion [19] and lower agreeableness [20] but inconsistent differences in neuroticism [19, 27] as well as no differences in any distinctive characteristics [23] compared with other specialties. Students specializing in psychiatry have shown lower conscientiousness compared with surgeons [20], and higher openness [21–23], neuroticism [23] and agreeableness [21] compared with other specialties.

However, the contribution of personality traits to performance and success in medicine is suggested to be greater in the practice of medicine than in medical education [18, 28]. Deciding to become a physician is an educational choice whereas selecting a specialty is seen as an occupational choice that reflects how personality types will interact with medical specialty work environments and how specialists will modify their practice of medicine within the specialty to better fit their personality [21, 28]. However, little is known about how personality traits influence and modify medical specialization and the career choices of physicians-in-practice *after* graduating from medical school when actually practicing and experiencing various medical specialties that differ in terms of requirements, work settings, routines, rewards and vocational interests [21, 29].

In addition, present research and future directions in the associations between personality traits and medical career selection suggest that the expression of trait-relevant behaviour as well as the predictive validity of personality traits for person-job fit in medicine is context dependent, having both costs and benefits which become evident in later careers and particularly in clinical practice [28, 30, 31]. This means that personality traits conventionally perceived as “good”, such as conscientiousness, also have a “dark-side”, and “bad” traits, such as neuroticism, have a “bright-side”, in terms of physicians’ clinical practice and well-being at work [28, 31]. For example, conscientiousness, which is found to be the most significant predictor of person-job fit in medical education [18], may be a valid predictor in clinical contexts where higher conscientiousness is expressible (e.g., a surgical operation) but less valid in contexts where it is not as expressible (e.g., patient interaction or clinical practice that demands flexibility) [28]. Similarly, too little neuroticism together with anxiety may hinder the acquisition of medical knowledge and skills and therefore also slow professional development [30]. These may have implications for a physician’s perceptions of person-job fit within the current specialty and further influence his/her career decision-making process and choices in terms of employment sector, amount and type of clinical patient work and potential change of specialty, for example [21].

Yet researchers and professionals in medical education have emphasized the importance of exploring the differential prediction of personality traits across the medical career and how personality traits influence specialists’ career choices and modifications in the long run when practicing a certain specialty [28, 30, 31]. Studies examining specialty residents in clinical practice have used small non-representative samples [27, 32–34] or have focused only on certain specialties such as surgery [9, 33, 35]. Moreover, whether personality traits contribute to important career choices such as working in the private or public sector, with or without clinical patient contact as well as change of specialty remains largely unexplored [18]. What is known at the general level is that work-related factors and practical scenarios associated with public versus private sectors have been found to be associated with the type and amount of clinical patient work that, in turn, might be found appealing by different personality types [18, 36, 37]. As far as we know, only one study has examined change of specialty choices and even then only among recently graduated students [5]. Junior doctors who had chosen psychiatry were found to be more likely to change their specialty than those who had chosen general practice [5]. The stability of their choice was not, however, related to personality and confidence or satisfaction with medicine in general, but instead to enjoyment and the lifestyle factors associated with the specialty. To sum up, then, an important gap exists in our understanding of the role of personality in medical specialists’ career choices in the long term for developing medical career counseling and interventions, and more explicitly, to help students and medical professionals choose medical careers that best fit their personality and individual preferences.

In a nationally representative sample of Finnish physicians (*N* = 2837) we examined how personality traits are associated with medical career choices *after* graduating from medical school, in terms of employment sector, patient contact, medical specialty and change of specialty.

## Methods

### Study design

A retrospective cross-sectional cohort study.

### Population and data collection

The data are from the ongoing longitudinal Finnish Health Care Professionals’ Study (HPS) [38, 39]. The HPS consists of baseline data collected in 2006, and two follow-up measurement points in 2010 and 2015. In 2006, 2010 and 2015, random samples of 5000, 7000 and 8374 Finnish physicians, respectively, were drawn from a database maintained by the Finnish Medical Association (FMA) [40] including all active licensed physicians in Finland. Each member of the samples received an e-mail invitation to participate in a Web-based survey followed by two reminder e-mails. A postal questionnaire was sent once to those who did not respond. The response rates were 57%, 55% and 50%, respectively. We included data from each measurement point (2006, 2010, 2015) only from physicians who had participated at least in 2015, and who had data for all study variables. HPS is representative of the eligible population in terms of gender, age and employment sector [38].

### Procedures and study hypotheses

We examined the associations between personality traits and different medical career choices in terms of employment sector (private *vs.* public), amount of clinical patient contact, specialty choice, and change of specialty, using a large nationally representative sample of currently active and licensed Finnish physicians representing 12 different categories of specialties. We also considered potential gender differences in personality traits suggested by previous research [13, 18, 23]. We expected to find distinctive personality traits associated with physicians’ career and specialty choices *after* medical school independent of known confounding factors within the specialty. As our study contains more population-based data with more frequent and specific categorizing of specialties compared with previous research recently conducted on the topic [19, 20, 22, 23, 27], and due to the inconsistency of findings from studies conducted among medical specialty residents *after* graduating from medical school [9, 27, 32, 33, 35], more specific hypotheses were not assessed.

### Database and definitions

#### Medical specialty

Medical specialty was self-reported in 2006, 2010 and 2015. First, respondents reported whether they were specialists or non-specialists. In Finland, a medical specialist degree requires five to six years of medical practice, including at least nine months of service in public health centres, theoretical and administrative courses, and a passing grade on a national written exam. Second, specialists were asked to report their specialty. If they had more than one specialty, they were advised to report their most recent. Third, 12 different specialties were categorized according to the official FMA classification [40]: (1) Anaesthesiology and Intensive Care Medicine, (2) Surgery (including all surgical sub-specialties), (3) Pediatrics (including Child neurology and Children’s disease), (4) Obstetrics and Gynecology, (5) Psychiatry (including Child Psychiatry, Adolescent Psychiatry, and Forensic Psychiatry), (6) Radiology, (7) Internal Medicine and Oncology, (8) Ophthalmology and Otorhinolaryngology, (9) Other specialties of internal medicine (e.g., Endocrinology, Gastroenterology, Dermatology and Allergology), (10) Occupational Health, and (11) General Practice, (12) Hospital Service Specialties (e.g., Clinical Microbiology, Forensic Medicine, Clinical Genetics). The most recent specialty was used in the analysis.

#### Personality

Personality traits were assessed in 2015 using the Five Factor Model of personality (FFM) [41, 42], the most established framework across different countries and cultures that examines normal adult personality traits [43]. The FFM consists of five personality dimensions: extraversion (referring to a tendency to be social, active and feel positive emotions), conscientiousness (referring to a tendency to be persistent, organized and achievement oriented), openness to experience (referring to a tendency to be curious, sensitive and open to variety), agreeableness (referring to a tendency to be trustful, cooperative and sympathetic), and neuroticism (referring to a tendency to be anxious and feel negative emotions such as fear and/or anger). We used the shortened 15-item version of the Big Five Inventory (S-BFI) [41], which consists of three items per personality trait assessed on a 5-point Likert scale ranging from 1 (totally disagree) to 5 (totally agree). Measurement reliability (Cronbach alpha; α) ranged from satisfactory to good; extroversion (α = .83), conscientiousness (α = .60), openness (α = .70), agreeableness (α = .52), and neuroticism (α = .79).

#### Gender, age, employment sector and clinical patient contact

Gender, age, employment sector, and patient work were self-reported in 2006, 2010 and 2015. The most recent value during intervals of the measurements was chosen. Employment sector was categorized as public (hospital, primary care, other municipal site of practice, state office or institution) or private (university, private practice, including private medical centres or clinics; foundation, association, or organization; and others, such as the pharmaceutical industry). When considered as a covariate, patient work was treated as a continuous variable referring to a physician’s self-reported weekly working hours with clinical patient contact. When treated as an outcome variable, patient work was indicated by four levels referring to a physician’s self-reported weekly working hours with clinical patient contact, and encoded as (0) no clinical patient contact (0 h per week), (1) some clinical patient contact (1–12 h per week), (2) clinical patient contact approximately half the weekly working time (13–26 h per week), and (3) clinical patient contact most of the weekly working time (27 h per week or more).

### Data analyses

All analyses were conducted using analyses of covariance (ANCOVA with posthoc tests for pairwise comparisons) adjusted for demographics (gender and age) and different combinations of confounding factors affecting physicians’ daily work (employment sector, clinical patient contact and medical specialty). Personality traits (extroversion, conscientiousness, openness to experience, agreeableness and neuroticism) were standardized (Mean = 0; Standard Deviation, SD = 1) and analysed separately with the probability value *p* < 0.05 set as a significance value level. All analyses were conducted with a StataMP 14.2 software package.

First, we examined gender differences in personality traits, adjusting for age, employment sector (public vs. private), specialty and clinical patient contact (hours per workweek treated as a continuous variable). Second, we investigated whether physicians’ gender would moderate the associations between personality traits and medical specialty choices after adjustment for age, employment sector and clinical patient contact. Personality traits by gender interactions for specialty choices were considered in each personality trait separately. As the associations between personality traits and specialty choices were not found to differ by physician’s gender, all subsequent analyses were conducted for women and men simultaneously.

Third, we examined the associations of personality traits with employment sector adjusted for gender, age, clinical patient contact (treated as a continuous variable) and specialty. Fourth, we examined the associations of personality traits with clinical patient contact (treated as a four-level categorical variable) adjusted for gender, age, employment sector and specialty. Fifth, we examined the associations of personality traits with medical specialty adjusted for gender, age, employment sector and clinical patient contact (treated as continuous variable).

Finally, we examined the associations of personality traits with change of specialty during intervals of the measurements, adjusted for gender, age, employment sector and clinical patient contact. For this purpose, specialists who responded the first time to the survey only in 2015 (*n* = 1650) were excluded from the analysis as they did not have the possibility to change their specialty by then. Also those who had missing data on some of the control variables (*n* = 201) were excluded from the analyses concerning the change of specialty.

### Ethics

The ethics committee of the Finnish National Research and Development Centre for Health and Welfare approved the study protocol. Informed consent was obtained from all study participants. As the data of the study were retrieved from a representative sample of an occupational cohort group with identification risks, the data were not made openly accessible. The data are, however, available on request by contacting Professor Marko Elovainio (marko.elovainio@helsinki.fi) and signing an official data user agreement.

## Results

Of 4145 physicians responding to the survey in 2015, 4005 provided information on all personality traits. Of these, we excluded 440 respondents who were not specialists and 728 specialists who had missing data on some of the study variables. Altogether, 2837 medical specialists formed the final sample representing 68.4% of the original sample.

The study sample included 2837 medical specialists (65% women) with a mean age of 49.4 years (Standard Deviation (SD) = 11.19; range 25 to 72 years). The characteristics of the study sample are presented in Table 1. Most of the medical specialists worked in the public sector (73%), and had clinical patient contact at least half or more (72%) of their weekly working time. General practice (19.8%) and other internal medicine specialties (15.3%) (e.g., Endocrinology, Gastroenterology, Dermatology and Allergology) formed the largest groups of specialists. Women represented the majority of respondents in all other specialties except surgery, where men were the most predominant (65.1%). Women specialists were also younger and more likely worked in the public employment sector than in the private sector compared with men specialists.

**Table 1:** Basic characteristics of 2837 Finnish physicians, by gender and specialty

	Women (*N* = 1838)		Men (*N* = 999)			Total (*N* = 2837)	
Characteristics	Number	Percent	Number	Percent	*p-value* ^a^	Number	Percent
Gender							
Women	1.838	65				1.838	65
Men			999	35		999	35
Age (M ± SD)	47.94 ± 10.63		52.02 ± 11.72		< 0.001	49.4 ± 11.19	
Employment sector					< 0.001		
Public	1393	67.59	668	32.41		2.061	73
Private	445	57.35	331	42.65		776	27
Medical specialty					< 0.001		
Anaesthesiology and Intensive Care Medicine	120	64.17	67	35.83		187	6.6
Surgery	80	34.93	149	65.07		229	8.1
Pediatrics	124	75.61	40	24.39		164	5.8
Obstetrics and Gynecology	155	83.33	31	16.67		186	6.6
Psychiatry	231	75.49	75	24.51		306	10.8
Radiology	55	52.88	49	47.12		104	3.6
Internal Medicine and Oncology	91	63.64	52	36.36		143	5.0
Ophthalmology and Otorhinolaryngology	68	51.91	63	48.09		131	4.6
Other specialties of Internal Medicine	270	62.21	164	37.79		434	15.3
Occupational Health	178	65.68	93	34.32		271	9.5
General Practice	403	71.84	158	28.16		561	19.8
Hospital Service Specialties	63	52.07	58	47.93		121	4.3
Clinical Patient Contact (hours per week; M ± SD)	18.74 ± 9.70		18.36 ± 11.14		0.36	18.61 ± 10.23	
No clinical patient contact	111	54.15	94	45.85		205	7.2
1–12 h per week	359	61.16	228	38.84		587	20.7
13–26 h per week	957	69.50	420	30.50		1.377	48.5
27 h per week or more	411	61.53	257	38.47		668	23.6
Personality trait (M ± SD; range 1–5)							
Extraversion	3.37 ± 0.90		3.16 ± 0.87		< 0.001	3.30 ± .89	
Conscientiousness	3.83 ± 0.75		3.62 ± 0.72		< 0.001	3.76 ± .75	
Openness to Experience	3.18 ± 0.74		3.30 ± 0.74		< 0.001	3.22 ± .75	
Agreeableness	3.40 ± 0.68		3.39 ± 0.67		0.83	3.39 ± .68	
Neuroticism	2.94 ± 0.84		2.62 ± 0.76		< 0.001	2.83 ± .82	

^a ^Categorical variables were compared by chi square tests and continuous variables were compared by two-sample t-tests

**Fig. 1:**
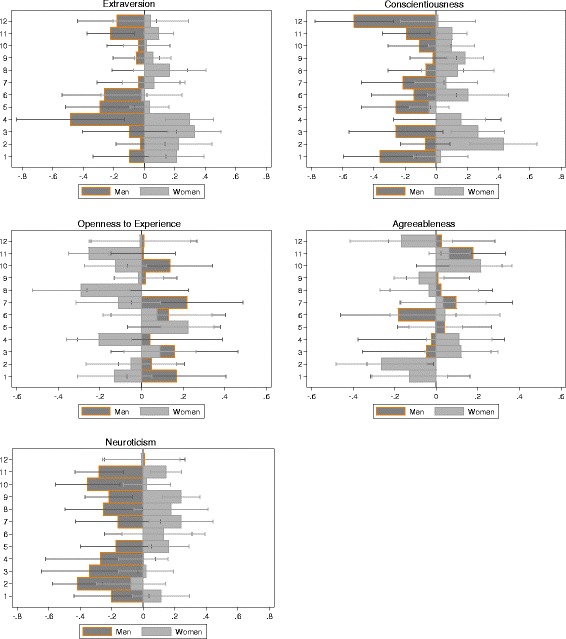
Finnish physicians’ (*N* = 2837) personality traits by specialty and gender. Units are standardized regression coefficients (β) and 95% confidence intervals. Analyses are adjusted for age, employment sector (private *vs.* public) and amount of clinical patient contact (hours per week). Specialty categories are: 1. Anaesthesiology and Intensive Care Medicine; 2. Surgery; 3. Pediatrics; 4. Obstetrics and Gynecology; 5. Psychiatry; 6. Radiology; 7. Internal Medicine and Oncology; 8. Ophthalmology and Otorhinolaryngology; 9. Other specialties of Internal Medicine; 10. Occupational Health; 11. General Practice; and 12. Hospital Service Specialties

Gender differences in personality traits by specialty (adjusted for age, employment sector and amount of clinical patient contact) are shown in Fig. 1. Women specialists scored higher in extraversion, conscientiousness and neuroticism but lower in openness compared to men specialists on average. No gender differences in agreeableness were observed.

The associations of personality traits with employment sector, amount of clinical patient contact, medical specialty and change of specialty are shown in Table 2. Specialists who worked in the private sector scored higher in openness and agreeableness compared with specialists working in the public sector. Specialists with clinical patient contact most of their working hours per week scored higher in conscientiousness but lower in openness compared to other specialists on average. Specialists with clinical patient contact approximately half of their working hours also had lower openness but higher neuroticism than other specialists. Furthermore, specialists who reported no clinical patient contact during their working hours had lower levels of extraversion but higher levels of openness compared with other specialists. No differences in any personality traits were found among specialists having some clinical patient contact, i.e., from 1 to 12 h per week.

**Table 2:** Associations of personality traits with employment sector ^a^, clinical patient work ^b^, and medical specialty ^c^ among 2837 Finnish physicians and with change of specialty ^d^among 986 Finnish physicians

Big 5 traits	Extraversion	Conscien-tiousness	Openness to Experience	Agreeableness	Neuroticism
Career variable	β (95% CI)	β (95% CI)	β (95% CI)	β (95% CI)	β (95% CI)
Employment sector^a^					
Public (*n* = 2061)	− 0.016 (− 0.07–0.03)	− 0.037 (− 0.09–0.01)	− 0.031*** (− 0.08–0.02)	− 0.045*** (− 0.09–0.01)	− 0.041 (− 0.09–0.01)
Private (*n* = 776)	0.070 (− 0.01–0.15)	− 0.001 (− 0.08–0.08)	0.122*** (0.04–0.20)	0.137*** (0.06–0.21)	− 0.118 (− 0.19- -0.04)
Clinical patient contact^b^					
No patient contact (*n* = 205)	− 0.089* (− 0.18–0.00)	− 0.059 (− 0.15–0.03)	0.090* (0.00–0.18)	0.017 (− 0.07–0.11)	− 0.009 (− 0.10–0.08)
Some patient contact (1–12 h/week; *n* = 587)	0.060 (− 0.01–0.13)	− 0.010 (− 0.08–0.06)	0.050 (− 0.02–0.12)	0.026 (− 0.04–0.10)	− 0.062 (− 0.13–0.01)
Patient contact ca half of the time (13–26 h/week; *n* = 1377)	0.002 (− 0.05–0.06)	− 0.004 (− 0.06–0.05)	− 0.060* (− 0.12- -0.00)	− 0.012 (− 0.07–0.04)	0.068 (0.01–0.12)
Patient contact most of the time (27 h/week or more; *n* = 668)	0.028 (− 0.04–0.10)	0.073* (0.01–0.14)	− 0.080* (− 0.15- -0.01)	− 0.031 (− 0.10–0.04)	0.002 (− 0.07–0.07)
Medical Specialty^c^					
Anaesthesiology and Intensive Care Medicine (*n* = 187)	0.057 (− 0.08–0.20)	− 0.125 (− 0.26–0.01)	− 0.011 (− 0.15–0.13)	− 0.109 (− 0.25–0.03)	0.011 (− 0.13–0.15)
Surgery (*n* = 229)	0.089 (− 0.04–0.22)	0.178** (0.52–0.30)	− 0.020 (− 0.15–0.11)	− 0.214** (− 0.34- -0.09)	− 0.184** (− 0.31- -0.06)
Pediatrics (*n* = 164)	0.156* (0.01–0.30)	0.090 (− 0.05–0.23)	0.135 (− 0.01–0.28)	0.084 (− 0.06–0.23)	− 0.098 (− 0.24–0.05)
Obstetrics and Gynecology (*n* = 186)	0.078 (− 0.06–0.22)	0.074 (− 0.06–0.21)	− 0.122*** (− 0.26–0.02)	0.096 (− 0.04–0.23)	− 0.102 (− 0.24–0.04)
Psychiatry (*n* = 306)	− 0.112* (− 0.22- -0.00)	− 0.146** (− 0.25--0.04)	0.235*** (0.12–0.34)	0.010 (− 0.10–0.12)	0.051 (− 0.06–0.16)
Medical Specialty^c^					
Radiology (*n* = 104)	− 0.146 (− 0.33–0.03)	0.059 (− 0.12–0.24)	0.094 (− 0.08–0.27)	− 0.068 (− 0.25–0.11)	0.136 (− 0.04–0.31)
Internal Medicine and Oncology (*n* = 143)	− 0.015 (− 0.17–0.14)	− 0.050 (− 0.20–0.10)	0.019 (− 0.13–0.17)	0.057 (− 0.10–0.21)	0.109 (− 0.04–0.26)
Ophthalmology and Otorhinolaryngology (*n* = 131)	0.091 (− 0.07–0.25)	0.060 (− 0.10–0.22)	− 0.166 (− 0.33- -0.01)	− 0.010 (− 0.17–0.15)	0.025 (− 0.14–0.19)
Other specialties of Internal Medicine (*n* = 434)	− 0.021 (− 0.12–0.07)	0.095* (0.00–0.19)	0.010 (− 0.08–0.10)	− 0.048 (− 0.14–0.05)	0.087 (− 0.01–0.18)
Occupational Health (*n* = 271)	− 0.047 (− 0.17–0.07)	0.008 (− 0.11–0.13)	− 0.011 (− 0.13–0.11)	0 .183** (0.06–0.30)	− 0.100 (− 0.22–0.02)
General Practice (*n* = 561)	− 0.054 (− 0.14–0.03)	− 0.017 (− 0.10–0.07)	− 0.157*** (− 0.24- -0.07)	0.098 (0.01–0.18)	0.010 (− 0.08–0.10)
Hospital Service Specialties (*n* = 121)	− 0.074 (− 0.24–0.10)	− 0.226** (− 0.39- -0.06)	− 0.006 (− 0.17–0.16)	− 0.080 (− 0.25–0.09)	0.054 (− 0.11–0.22)
Change of Specialty^d^					
Specialty not changed (*n* = 893)	0.015* (−0.06–0.08)	0.029 (− 0.04–0.10)	0.040* (− 0.03–0.11)	0.068 (− 0.00–0.14)	− 0.110 (− 0.18- -0.04)
Specialty changed (*n* = 93)	0.242* (0.04–0.44)	0.065 (− 0.14–0.27)	0.256* (0.05–0.46)	0.264 (0.06–0.47)	− 0.295 (− 0.49- -0.10)

*Note.* The results are based on analyses of covariance with posthoc tests for pairwise comparisons. β = Standardized regression coefficient (Mean = 0, SD = 1). 95% CI = 95% confidence interval for Exp (β). *p < 0.05: **p < 0.01; ***p < 0.001. Scores for personality traits range from 1 to 5, with higher scores indicating higher levels of the trait

^a^Adjusted for gender, age, clinical patient contact (hours per week; treated as a continuous variable) and specialty. Physicians working in the public sector serve as a reference group

^b^Adjusted for gender, age, employment sector (public vs. private) and specialty

^c^Adjusted for gender, age, employment sector and clinical patient contact

^d^Adjusted for gender, age, employment sector and clinical patient contact (hours per week; treated as a continuous variable). Physicians who did not change their specialty serve as a reference group. Specialists who responded the first time to the survey only in 2015 (*n* = 1650) and who had missing data on some of the control variables (*n* = 201) were excluded from the analyses

^b, c, d^The contrasts between groups are based on the standardized average mean (with a mean of zero and a standard deviation of one) of all specialists who form a reference group

Pediatricians showed higher extraversion whereas psychiatrists showed lower extraversion and conscientiousness but higher openness compared to other specialists on average. Surgeons showed higher conscientiousness but lower agreeableness and neuroticism than other specialists. Also, specialists from the other sub-specialties of internal medicine (e.g., Endocrinology, Gastroenterology, Dermatology and Allergology) scored higher in conscientiousness whereas specialists from the hospital service specialties (e.g., Clinical Microbiology, Forensic Medicine, Clinical Genetics) scored lower in conscientiousness compared to other specialists. Ophthalmologists and otorhinolaryngologists as well as general practitioners showed lower openness compared to other specialists. Specialists in occupational health and general practitioners showed higher agreeableness than other specialists.

Specialists who reported having changed their specialty showed higher extraversion and openness compared to specialists who had not changed specialty during the measurement intervals of the current study.

## Discussion

The present study using a nationally representative sample of Finnish physicians showed that of five major personality traits, openness was the most consistent trait associated with physicians’ career choices. Higher openness was associated with working in the private sector, not having clinical patient contact, specializing in psychiatry and having a tendency to change specialty. Lower openness, in turn, was associated with a high or average amount of clinical patient contact and specializing in general practice as well as in ophthalmology and otorhinolaryngology.

Of the other personality traits, higher conscientiousness was associated with a higher amount of clinical patient contact and specializing in surgery and in other internal medicine specialties whereas lower conscientiousness was associated with specializing in psychiatry and in hospital service specialties. Higher agreeableness was associated with working in the private sector and specializing in general practice as well as occupational health service whereas lower agreeableness was associated with specializing in surgery. Higher neuroticism was associated with an average amount of clinical patient contact whereas lower neuroticism was associated with specializing in surgery. Women scored higher in extraversion, conscientiousness and neuroticism but lower in openness compared with men. Specialists with no clinical patient contact showed lower extraversion whereas higher extraversion was associated with specializing in pediatrics and psychiatry and a change of specialty.

The association of higher openness with working in the private sector and changing specialties is understandable, as openness facilitates acceptance, flexibility and adequate adjustment to situational changes [41]. Openness has been linked to academic ability and divergent thinking [44, 45] and is seen as becoming more beneficial particularly in clinical education and in the applied settings of medicine [18, 46] than in academic achievement during medical education [47, 48]. Researchers use the expression “getting along” as a reflection of the “Openness to experience” personality trait, which seems to facilitate the optimal interpersonal interaction between a physician and a patient [18, 49, 50]. Obviously, these kinds of attributes may also be beneficial when working in the private sector in close, intense and permanent patient relationships and/or needing to change either employment sector or specialty during a medical career. Psychiatrists’ greater openness in the present study is also consistent with previous research [21–23]. Psychiatrists may benefit in their work from being more open, as this characteristic also reflects general attentiveness to inner feelings, intellectual curiosity and independence of judgment [41]. Specialists with no clinical patient contact showed higher openness, which may refer to hospital-based or procedure-oriented specialists and/or basic applied laboratory researchers with minimal patient contact. Compared with other specialties, the job description of medical researchers, for example, may also allow and make it generally easier to show intellectual curiosity and divergent thinking, thus reflecting higher openness to experience [41, 42].

Lower openness was associated with a high or average amount of clinical patient contact and specializing in general practice as well as in ophthalmology and otorhinolaryngology. The results may refer to physicians being greatly responsible for patients in general. General and family practitioners have been characterized as strict followers of clinical guidelines and principles compared to surgeons, for example [33]. Family practitioners have been found to be mixed in openness compared with other specialists, and, in any case, score lower on openness compared with psychiatrists and surgeons [18, 21]. Also, ophthalmologists and otorhinolaryngologists have been categorized as specialists having a more controlled lifestyle [9] reflecting lower openness. The job description in general practice as well as in ophthalmology and otorhinolaryngology is based on relatively traditional rules and regular operations [9]. Therefore, physicians representing these specialties may benefit from their lower openness. As well, stability of choice of specialty has been found to be significantly higher among general practitioners compared to psychiatrists, for example [5].

Higher conscientiousness was associated with high amounts of clinical patient contact and specializing in surgery and in other internal medicine specialties whereas lower conscientiousness was associated with specializing in psychiatry and in hospital service specialties. Conscientiousness has been found to be the best predictor of academic success in both preclinical and clinical phases of medical education [14, 18, 32, 46]. In a recent study among Swedish doctors, psychiatrists scored lower on conscientiousness particularly when compared with surgeons [20]. Surgeons’ higher tendency to be organized, careful and persistent is perceived as the most supportive characteristic considering the requisite skills of the surgical specialty [21, 33, 46]. Internists (including many subspecialties of internal medicine), in turn, have been suggested to score higher on conscientiousness because of their high self-reliance [21]. In addition, hospital service physicians showed lower conscientiousness compared with surgeons in the above-mentioned Swedish study [20].

Higher neuroticism was associated with an average amount of clinical patient contact whereas lower neuroticism was associated with specializing in surgery. The results are completely new not previously demonstrated. Neuroticism has been found to be predictive of jobs and working environments where employees work in groups [41]. Individuals with higher neuroticism have been shown to experience life events more negatively than other individuals [51] partly because they choose to place themselves in situations that foster negative effects [52]. Taking into account that medicine is an emotionally demanding field [53], this trait might have repercussions on physicians’ well-being indicators such as perceived stress and job satisfaction [54]. However, neuroticism has been shown to be rarely apparent among medical specialists in general [21]. Distinctive differences in neuroticism have been found only in a small cross-sectional study where surgeons scored at the highest level in neuroticism compared with other specialists [19]. In a study by Hoffman and colleagues, surgeons scored lower in neuroticism compared with the general population but not when compared with medical students or with other medical specialists [27]. Our results, however, suggest that the challenging, risk-taking and meticulous nature of surgical specialization may attract and favour physicians who do not have a general tendency to experience negative emotions in response to stressful duties and situations [41].

Higher agreeableness was associated with working in the private sector and specializing in general practice as well as in occupational health whereas lower agreeableness was associated with specializing in surgery. Agreeableness has been found to be predictive of clinical competence in medical students [55, 56], suggesting that it may facilitate physician-patient relationships [18]. Previously, general practice (including family medicine) and occupational health have been classified as person-oriented specialties whose physicians show sympathetic, trusting and cooperative behaviour, reflecting higher Agreeableness [21, 33]. Surgeons’ higher tendency to be demanding, dominant and tough-minded, referring to their lower agreeableness, is consistent with previous research [20, 21, 33, 46].

Specialists who did not have clinical patient contact showed lower extraversion whereas higher extraversion was associated with specializing in pediatrics and change of specialty. The job description of medical researchers with no clinical patient contact, for example, may allow and make it easier to show withdrawal and deliberate behaviour in general. Higher extraversion, in turn, reflects approaching behaviour and general sociability with a cheerful disposition [41], which can be seen as favourable characteristics of physicians working with children and encountering new working environments. Although psychiatrists also scored slightly higher on extraversion compared with other specialists, they may vary within the specialty concerning this trait with respect to trait-related single facets such as being sociable and outgoing [21]. Doctors choosing psychiatry have been found to be more likely to change their specialty than those choosing general practice [5]. Choice stability has not, however, been previously related to personality and confidence, or to satisfaction with medicine in general, but instead to the job satisfaction and lifestyle factors associated with the specialty [5].

Women specialists scored significantly higher in extraversion, conscientiousness and neuroticism but lower in openness compared to men physicians. Gender differences in agreeableness or gender by specialty interactions, in any personality trait, were not observed. Women physicians have previously been found to score higher also in agreeableness whereas gender differences with regard to neuroticism have not been shown [23]. Gender-related individual-level factors such as personality might be a considerable variable to take into account in career counseling and specialty guidance during medical education [18, 57] in order to enhance person-job fit [16] among physicians.

### Strengths and limitations

The greatest strength of this study is the relatively large and representative population-based sample of actively working licensed Finnish physicians [38, 39], an important advantage compared with previous research on the topic. Second, as far as we know, the present study is the first to demonstrate the significant role of physicians’ personality traits regarding medical career including chosen specialty, employment sector, involvement in clinical patient work and change of specialty *after* medical education when actually practicing different specialties that differ in requisite skills, job duties, work settings and vocational interests. Third, the associations of physicians’ personality traits with the amount of clinical patient contact and change of specialty are new findings. Similarly, the findings regarding surgeons’ lower neuroticism and occupational health specialists’ higher agreeableness are novel. The findings concerning openness and agreeableness, in particular, might have practical relevance in physician-patient interpersonal relationships. These new findings of the current study may be useful when developing medical career counseling and interventions during and after medical education to help students choose medical careers that best suit their individual personalities.

The present study has limitations as well. The possibility of reverse causality cannot be ruled out. Although personality traits are moderately heritable [58] and relatively stable over the life course [17, 59], major life-events have been found to affect personality development [60–63]. Thus, it is also possible that a physician’s personality would be affected as a result of medical education and/or chosen career [28]. As well, the missing data more than 10% of the participants may have introduced potential bias in parameter estimation, thus weakening the generalizability of the results. The physicians’ personality traits were measured only in 2015 at one follow-up point of the study. Longer-term follow-ups would be necessary to gain a larger and more reliable picture of how a physician’s personality contributes to career choices, and to shed light on questions of causality.

Furthermore, we cannot rule out the possibility of residual confounding. Other factors (e.g., job satisfaction and experiences during and/or after medical education) may explain the link between physician personality and career choice. Although carried out with the permission of the FMA [40], the study was based on voluntary participation. Therefore, the self-selection of physicians participating in the study may explain some of the results.

To assess personality, we used the well-known shortened version of the S-BFI [41] that has been found to have adequate reliability and convergent validity [42, 64] also among medical students [46]. Despite the obvious advantages of short personality measures found for research purposes [64], shortened scales might not completely cover all the delicate facets of the FFM. Although only satisfactory in agreeableness, the reliability coefficients of the scales were consistent with previous research conducted with the S-BFI [42, 64].

## Conclusions

The results showed distinctive personality traits to be associated with physicians’ career and specialty choices *after* medical school independent of known confounding work-related factors. Openness was the most consistent personality trait associated with physicians’ career choices in terms of employment sector, amount of clinical patient contact, specialty choice and change of specialty. The findings concerning openness and agreeableness, in particular, might have practical relevance in the interpersonal physician-patient relationship. Our results also suggest that gender-related personality might be a considerable individual-level factor to take into account in career counseling and specialty guidance during and after medical education in order to enhance the person-job fit of physicians. Whether and how a physician’s personality is related to job satisfaction and general well-being within the chosen specialty, including employment sector, clinical patient work and change of specialty, is an unexamined and important topic for future research.

## Abbreviations

ANCOVA: Analysis of Covariance
FFM: Five Factor Model of Personality
FMA: Finnish Medical Association
HPS: Health Professionals Study
S-BFI: Shortened version of Big Five Inventory
SES: Parental Socioeconomic Status
